# A Practical Treatise on the Diseases of Children

**Published:** 1848-09

**Authors:** 


*A Practical Treatise on the Diseases of Children.* By J. Forsyth Meigs, M. D., Lecturer on the Diseases of Children in the Philadelphia Medical Association, Fellow of the College of Physicians of Philadelphia. 12mo. pp. 575. Lindsay & Blakiston. Philadelphia: 1848.

This work is the third of the series of Manuals in the course of publication by one of our enterprising Philadelphia publishing houses. The two first we have already noticed in terms of commendation, and it is but just to state that the present is entirely on a level with its predecessors. The author is a son of the eminent Professor of Obstetrics in Jefferson Medical College, and, like him, is distinguished for his ardent devotion to obstetrics, and to the study and treatment of the diseases of women and children.

"The motives," we are told, "which led the author of this volume of the Medical Practitioner's and Student's Library, to undertake its preparation, were the hope that the details of his own experience might prove of some utility, and the belief that a work on the Diseases of Children, executed upon a somewhat different plan from those already before the profession, might not be an unacceptable addition to the medical literature of the country."

"In the preparation of the work no pains has been spared to make it both methodical and accurate, and as complete as the limits of the series would allow. The classification of diseases according to the systems which they affect, has been adopted by the writer as the most convenient. The divisions of each article are those employed by the most eminent among recent systematic writers."

Although the preface announces as a prominent motive of the author, in preparing this volume, to give "the details of his own experience" to the profession, still, he acknowledges that he "has availed himself, as fully as possible, of every authority of importance placed within his reach, always, however, endeavouring to judge what came under his notice, by knowledge derived from his personal experience in private practice." In the expressive language of Dr. Bell, we may say that the author "disclaims the particular absurdity of professing to write an original work on the practice of medicine."

The diseases which are treated of in the work, are comprised in five CLASSES: Class I. Treats of *Diseases of the Respiratory Organs.* Class II. *Diseases of the Digestive Organs.* Class III. *Diseases of the Nervous System.* Class IV. *Eruptive Fevers.* Class V. *Worms in the Alimentary Canal.* These again are further divided, according as they are marked or not by appreciable alterations of structure.

It will be perceived that all the diseases to which infancy and childhood are particularly predisposed, are not included in this classification. Thus we have nothing on diseases of the circulation, nor diseases of the skin, except eruptive fevers; etc. These omissions, we presume, are in consequence of the limited space assigned to the volume; but we cannot help expressing the hope that it will be otherwise in the next edition. Upon this point, we would call the attention of our friend, the author, to the opinion of Dr. Johnson, that "a writer is required to make his book a whole, which shall contain all the reader wishes to be informed of upon the subject laid before him." The general merits of the book entitle it to be thus perfected. As it is, the production is creditable to the industry and discrimination of the author, and well calculated as a text-book for students, or for ready reference by the young practitioner. The reader will find it to contain, in a brief compass, the most approved views of modern authoritative writers, expressed in plain and appropriate language.

